# Supplementary material for: Comparative Use of Contralateral and Sham-Operated Controls Reveals Traces of a Bilateral Genetic Response in the Rat Brain after Focal Stroke
Source: Int J Mol Sci. 2022 Jun 30;23(13):7308. doi: 10.3390/ijms23137308 (PMC9266805; doi:10.3390/ijms23137308)
Supplement: Supplementary file 1 [file ijms-23-07308-s001.zip › Supplementary Figure S1.pptx]

## Slide 1
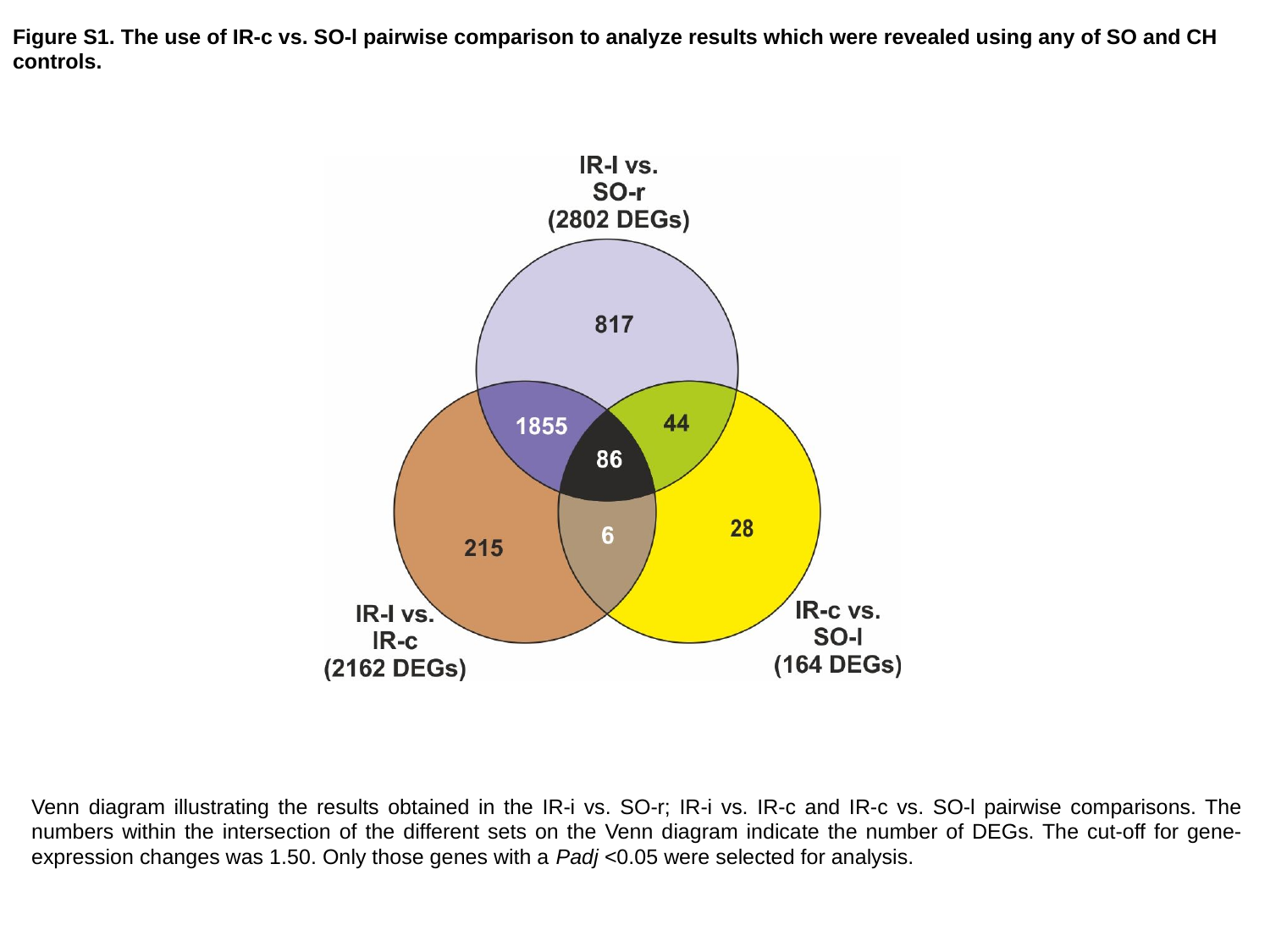

Figure S1. The use of IR-c vs. SO-l pairwise comparison to analyze results which were revealed using any of SO and CH controls.
Venn diagram illustrating the results obtained in the IR-i vs. SO-r; IR-i vs. IR-c and IR-c vs. SO-l pairwise comparisons. The numbers within the intersection of the different sets on the Venn diagram indicate the number of DEGs. The cut-off for gene-expression changes was 1.50. Only those genes with a Padj <0.05 were selected for analysis.
