# Supplementary material for: Comparative Use of Contralateral and Sham-Operated Controls Reveals Traces of a Bilateral Genetic Response in the Rat Brain after Focal Stroke
Source: Int J Mol Sci. 2022 Jun 30;23(13):7308. doi: 10.3390/ijms23137308 (PMC9266805; doi:10.3390/ijms23137308)
Supplement: Supplementary file 1 [file ijms-23-07308-s001.zip › Supplementary Figure S2.pptx]

## Slide 1
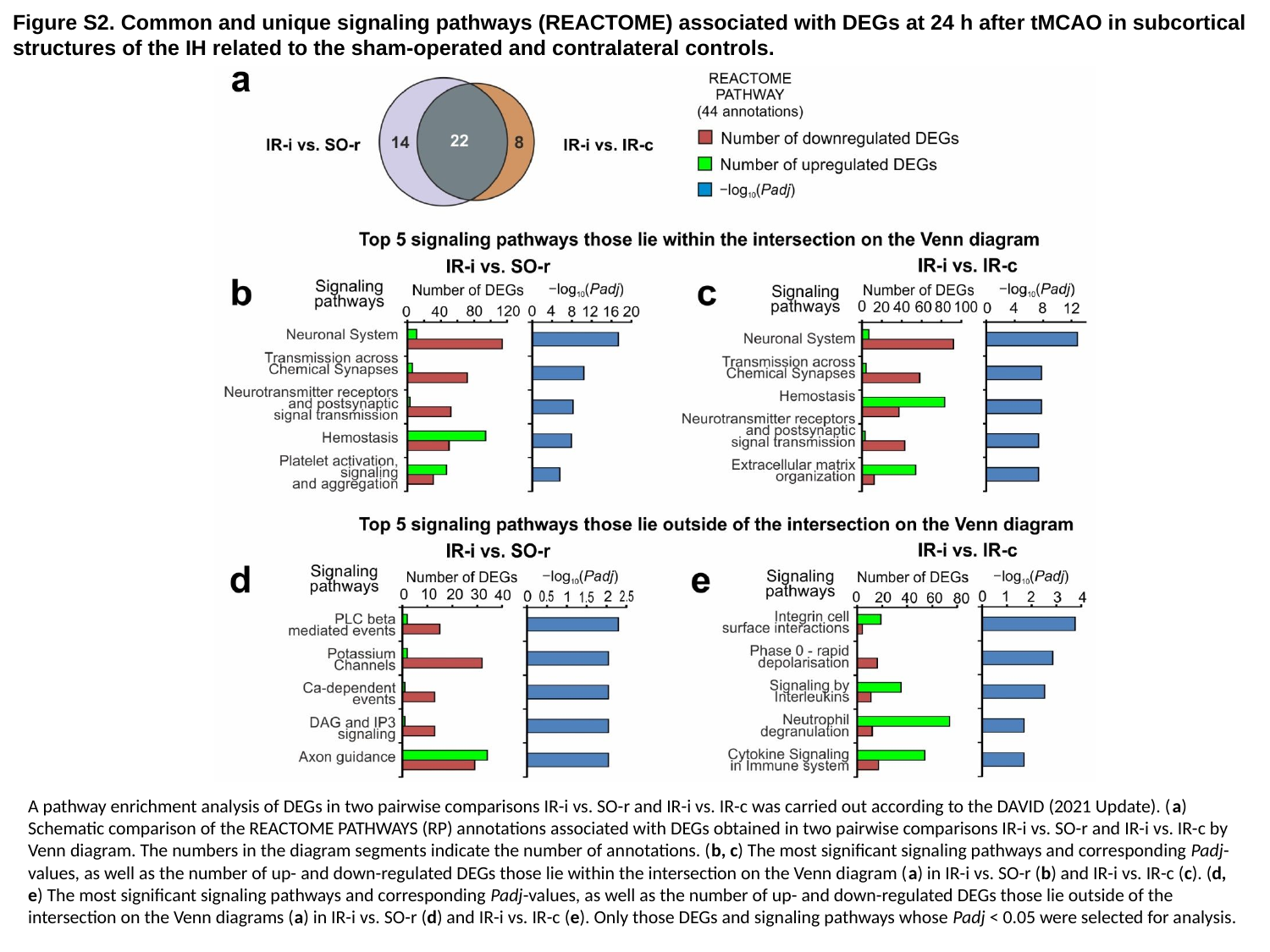

Figure S2. Common and unique signaling pathways (REACTOME) associated with DEGs at 24 h after tMCAO in subcortical structures of the IH related to the sham-operated and contralateral controls.
A pathway enrichment analysis of DEGs in two pairwise comparisons IR-i vs. SO-r and IR-i vs. IR-c was carried out according to the DAVID (2021 Update). (a) Schematic comparison of the REACTOME PATHWAYS (RP) annotations associated with DEGs obtained in two pairwise comparisons IR-i vs. SO-r and IR-i vs. IR-c by Venn diagram. The numbers in the diagram segments indicate the number of annotations. (b, c) The most significant signaling pathways and corresponding Padj-values, as well as the number of up- and down-regulated DEGs those lie within the intersection on the Venn diagram (a) in IR-i vs. SO-r (b) and IR-i vs. IR-c (c). (d, e) The most significant signaling pathways and corresponding Padj-values, as well as the number of up- and down-regulated DEGs those lie outside of the intersection on the Venn diagrams (a) in IR-i vs. SO-r (d) and IR-i vs. IR-c (e). Only those DEGs and signaling pathways whose Padj < 0.05 were selected for analysis.
