# Supplementary material for: Comparative Use of Contralateral and Sham-Operated Controls Reveals Traces of a Bilateral Genetic Response in the Rat Brain after Focal Stroke
Source: Int J Mol Sci. 2022 Jun 30;23(13):7308. doi: 10.3390/ijms23137308 (PMC9266805; doi:10.3390/ijms23137308)
Supplement: Supplementary file 1 [file ijms-23-07308-s001.zip › Supplementary Method S1.pptx]

## Slide 1
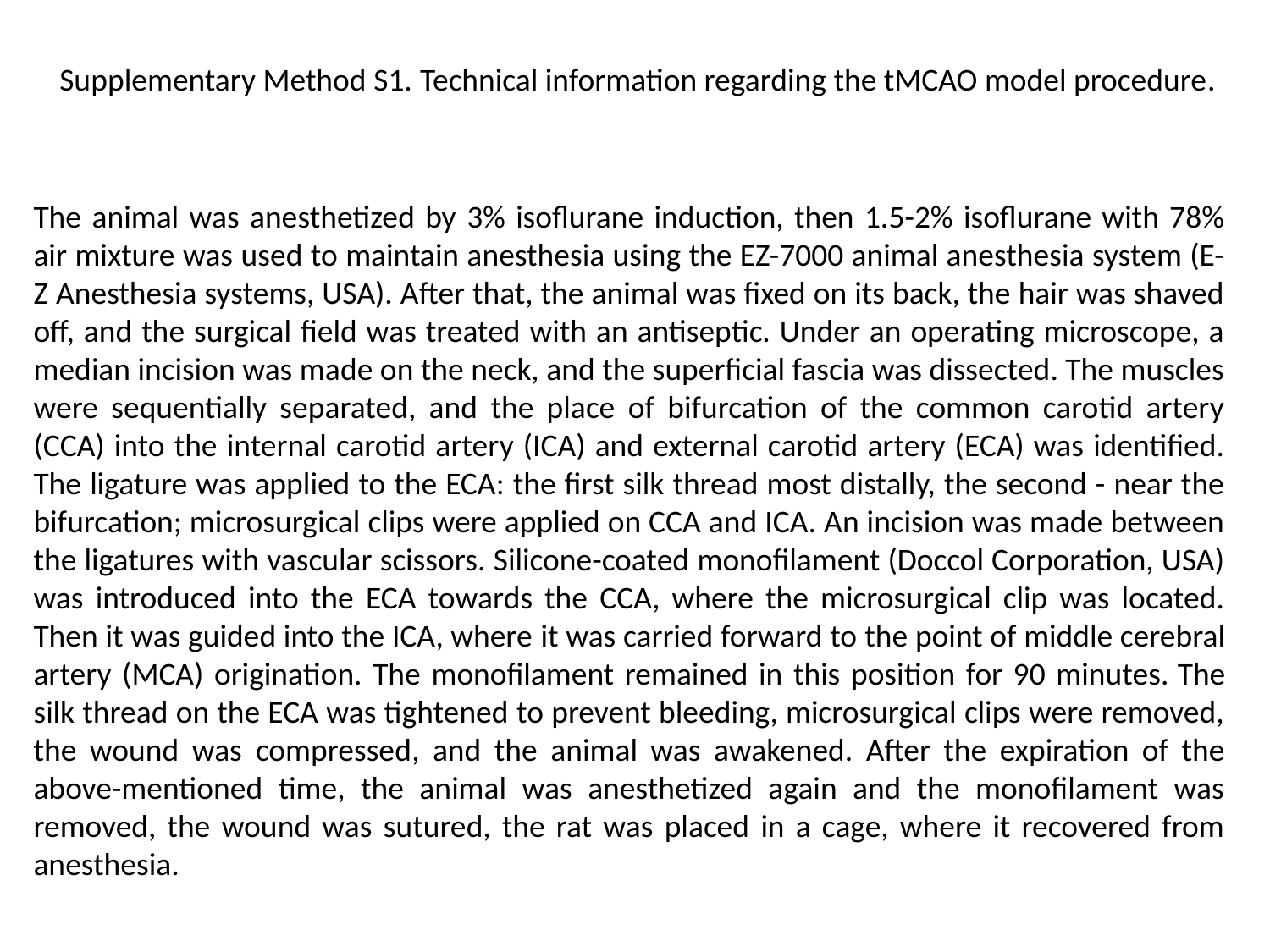

Supplementary Method S1. Technical information regarding the tMCAO model procedure.
The animal was anesthetized by 3% isoflurane induction, then 1.5-2% isoflurane with 78% air mixture was used to maintain anesthesia using the EZ-7000 animal anesthesia system (E-Z Anesthesia systems, USA). After that, the animal was fixed on its back, the hair was shaved off, and the surgical field was treated with an antiseptic. Under an operating microscope, a median incision was made on the neck, and the superficial fascia was dissected. The muscles were sequentially separated, and the place of bifurcation of the common carotid artery (CCA) into the internal carotid artery (ICA) and external carotid artery (ECA) was identified. The ligature was applied to the ECA: the first silk thread most distally, the second - near the bifurcation; microsurgical clips were applied on CCA and ICA. An incision was made between the ligatures with vascular scissors. Silicone-coated monofilament (Doccol Corporation, USA) was introduced into the ECA towards the CCA, where the microsurgical clip was located. Then it was guided into the ICA, where it was carried forward to the point of middle cerebral artery (MCA) origination. The monofilament remained in this position for 90 minutes. The silk thread on the ECA was tightened to prevent bleeding, microsurgical clips were removed, the wound was compressed, and the animal was awakened. After the expiration of the above-mentioned time, the animal was anesthetized again and the monofilament was removed, the wound was sutured, the rat was placed in a cage, where it recovered from anesthesia.
